# Supplementary material for: Feeding Behavior, Gut Microbiota, and Transcriptome Analysis Reveal Individual Growth Differences in the Sea Urchin Strongylocentrotus intermedius
Source: Biology (Basel). 2024 Sep 7;13(9):705. doi: 10.3390/biology13090705 (PMC11428599; doi:10.3390/biology13090705)
Supplement: Supplementary file 1 [file biology-13-00705-s001.zip › biology-3135985-supplementary.pdf]

After 16S rRNA sequencing, the results showed that a total of 591,184 optimized sequences were acquired, comprising a total of 244,583,486 bp, and possessing an average sequence length of 413.983987 bp, These results met the sequencing specifications of the Humina Miseq PE300 platform (Table S1).

Table S1. 16S rRNA sequencing quality control and Effective Tags.

**Sample Name**	**Effective Tags**	**Base number/bp**	**Average sequence length/bp**
L1	61,126	31,311,139	418
L2	69,371	34,371,177	406
L3	43,472	23,943,043	408
M1	53,855	32,888,008	421
M2	40,726	26,669,270	414
M3	80,363	35,405,451	406
S1	71,608	33,690,956	408
S2	65,048	29,573,940	408
S3	53,349	22,866,930	405
Total	538,918	270,719,914	411

Transcriptome sequencing obtained a total of 390,605,952 raw data, of which the count of clean reads amounted to 383,668,490, with a clean read ratio reaching 98.22%. Subsequent filtering of the sequencing results revealed that the proportion of Q20 bases exceeded 96%, while the proportion of Q30 bases exceeded 91%, and the GC content ranged from 36.97% to 40.19%. The integrity of the data is notably high, facilitating subsequent analyses upon successful quality inspection (Table S2).

Table S2. Sequencing data statistics table.

**Sample**	**Raw reads**	**Clean reads**	**Error rate**	**Q20(%)**	**Q30(%)**	**GC Content(%)**
L_1	47347386	46431394	0.03	96.87	91.69	36.97
L_2	43968366	43027688	0.03	97.01	92.02	39.08
L_3	42708628	41962330	0.03	96.86	91.76	40.19
M_1	44999082	44217870	0.03	97.02	92.03	39.42
M_2	42376220	41584576	0.03	96.82	91.68	39.37
M_3	41486102	40745306	0.03	97.13	92.30	39.17
S_1	41925750	41069156	0.03	96.99	91.93	36.66
S_2	41928478	41464734	0.03	96.95	91.81	38.28
S_3	43865940	43165436	0.03	97.07	92.07	38.67

Examination of the dilution curve reveals that as the sequencing depth increases incrementally, both sets of dilution curves exhibit a trend of flattening (Fig. S1), suggesting that the quantity of sample sequencing data adequately encompasses all bacterial group compositions, and consequently, the sequencing data results are highly reliable.

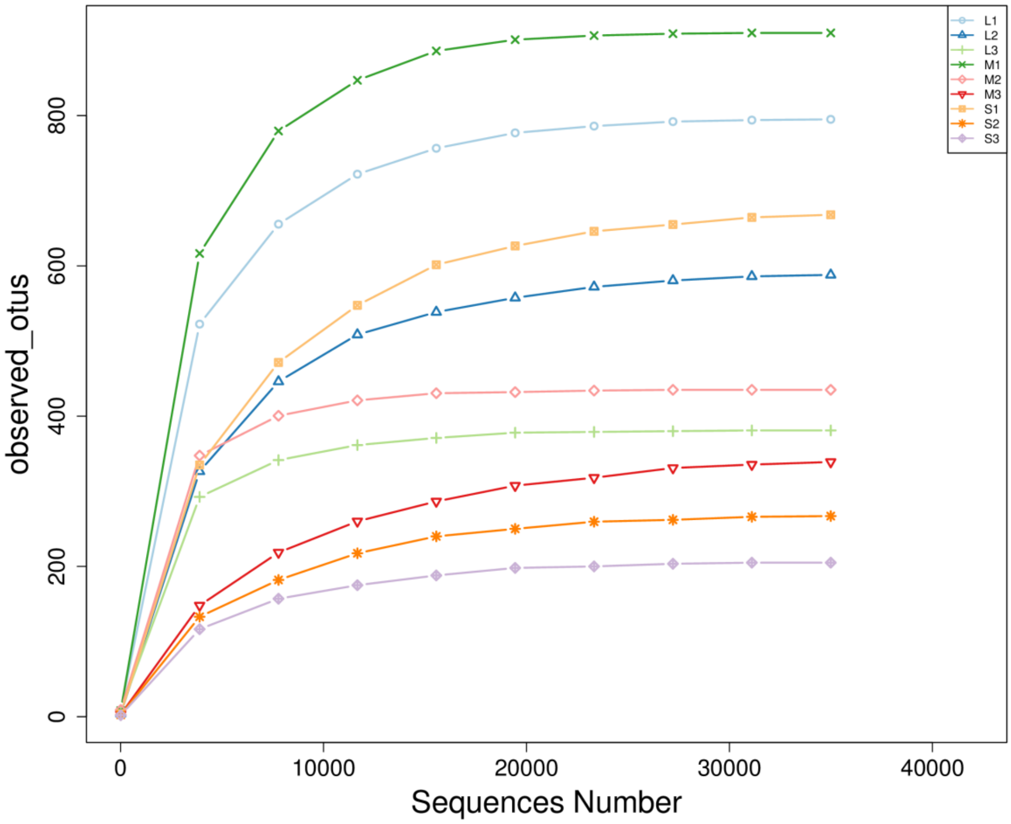

Figure S1. OTU dilution curve.
